# A technique of TA-assisted ILM peeling for myopic foveoschisis

**DOI:** 10.1186/s12893-025-03392-9

**Published:** 2025-12-11

**Authors:** Liyan Ye, Luyun Liang, Xiaolan Liu, Xiaohua Zhu, ZhongPing Chen, Yiqin Duan

**Affiliations:** 1 JiNan University, No. 601, West Huangpu Avenue, Tianhe District, Guangzhou City, Guangdong Province 510632 China; 2 Aier Eye Hospital Group Changsha Aier Eye Hospital, No. 188, Section 1, Furong South Road, Changsha City, Hunan Province 410000 China

**Keywords:** Internal limiting membrane peeling, Foveal-sparing, Myopic foveoschisis, Triamcinolone acetonide, Indocyanine green

## Abstract

**Objective:**

To evaluate the clinical effects of using Triamcinolone Acetonide (TA) in fovea-sparing internal limiting membrane peeling (FILMP) technique for treating myopic foveoschisis (MF).

**Methods:**

This prospective study recruited 66 (72 eyes) non-consecutive patients diagnosed with MF from June 2018 to November 2020. All patients underwent FILMP combined with 25G pars plana vitrectomy (PPV). Based on the staining agents used during FILMP, patients were divided into the TA group and the indocyanine green (ICG) group. Preoperative and follow-up assessments included visual acuity, best-corrected visual acuity (BCVA), intraocular pressure, slit-lamp examination of the anterior and posterior segments, optical coherence tomography (OCT), ultra-widefield fundus photography, and B-scan ultrasonography. Postoperative visual acuity, central retinal thickness (CRT), postoperative recovery of MF, and the incidence of postoperative full-thickness macular hole (MH) were compared between the two groups.

**Results:**

The mean age of patients was 56.16 ± 11.00 years (19 males and 47 females); the mean axial length was 30.50 ± 1.96 mm and the mean follow-up duration was 13.89 ± 8.32 months. Six patients underwent binocular surgery due to bilateral MF, while the remaining 60 patients underwent monocular surgery. Forty-four eyes received TA-assisted FILMP, and the remaining 28 eyes received ICG-assisted FILMP. A total of 65 patients, accounting for 90.3% (26 patients in the ICG group and 39 patients in the TA group), were treated with a 20% C3F8 tamponade. 40 patients, accounting for 69.4% (23 patients in the ICG group and 27 patients in the TA group), received combined 20% C3F8 tamponade and Phaco + IOL implantation. The total incidence of the postoperative full-thickness MH with FILMP was 4.9%. The mean postoperative logMAR visual acuity significantly improved compared to preoperative values (1.00 ± 0.62 vs. 1.67 ± 0.67, *p* < 0.01). Postoperative CRT was significantly thinner than the preoperative CRT (295.88 ± 167.55 μm vs 473.47 ± 195.96 μm, *p* < 0.01). There were no significant differences between the TA and ICG groups in the postoperative incidence of full-thickness MH, logMAR visual acuity, CRT, or MF recovery.

**Conclusion:**

Due to the strong adhesion of the posterior cortical vitreous, the internal limiting membrane (ILM) is difficult to stain with ICG. In contrast, TA can adhere well to the vitreous cortex and remain on the macular surface in eyes with MF. TA-assisted FILMP is a feasible approach for ILM peeling in MF. Large-sample, prospective and randomized controlled trials are needed to confirm these findings.

**Trial registration:**

Chinese Clinical Trial Registry, ChiCTR2200060265, 2022–05-24, Retrospectively registered.

## Introduction

Myopic foveoschisis (MF), a sight-threatening complication of high myopia, is characterized by intraretinal splitting between the thin outer retinal layer and the thick inner retinal layer [1]. The reported prevalence of MF in high myopic eyes ranges from 14.7% to 20% [2]. The combined effects of posterior staphyloma, vitreomacular adhesion, and epiretinal membrane (ERM) are potentially associated with MF progression. Pathological evidence shows that tangential traction on the internal limiting membrane (ILM) is a key contributing factor in the development of MF [3, 4]. As MF advances, complications such as macular hole (MH) and retinal detachment may occur [5].

Currently, no consensus exists regarding the optimal timing of surgery for MF. Surgery may be considered when patients present with symptoms such as decreased visual acuity or metamorphopsia [6]. Pars plana vitrectomy (PPV) combined with ILM peeling has been demonstrated to be an effective approach for managing MF [7, 8], as it relieves tangential traction in the macular region that might otherwise exacerbate the condition. However, complete peeling of the ILM at the fovea may disrupt the thin cystic wall, increasing the risk of complications. Studies have reported that the incidence of postoperative full-thickness MH and retinal detachment after ILM peeling ranges from 9.0% to 20.0% [5, 9]. To reduce the risk of postoperative MH, Shimada et al. proposed a modified technique known as fovea-sparing ILM peeling (FILMP) [10]. Lots of evidence indicated that FILMP can effectively reduce the occurrence of postoperative MH [11, 12].

Indocyanine green (ICG) has long been used to facilitate ILM visualization. However, its long-term safety of intravitreal administration remains unclear. Several studies have documented a tendency of ICG to accumulate in optic nerve tissues and retinal pigment epithelium (RPE), which may induce drug toxicity and lead to RPE atrophy [9]. Triamcinolone acetonide (TA) is commonly used to visualize the vitreous during the PPV. TA readily adheres to the posterior cortical vitreous and remains on the ILM surface. Using TA to assist ILM peeling may help avoid the potential retinal toxicity associated with ICG, although the technique requires a highly experienced surgeon. Brilliant Blue G (BBG) selectively stains the ILM and facilitates its visualization during peeling. Enaida et al. demonstrated that BBG selectively stains the ILM without significant toxicity, supporting its utility in chromovitrectomy [13]. However, Balaiya et al. suggested that the toxicity of BBG is related to both exposure time and concentration: excessive concentration or prolonged staining duration may still cause structural or functional retinal damage [14]. While BBG is effective for selectively staining the ILM, its efficacy may be limited in highly myopic eyes due to the complex vitreoretinal interface [15], including a tightly adherent posterior vitreous cortex and a thin ILM. In such cases, adjunctive visualization with agents such as TA may be advantageous.

Here, we evaluated the clinical efficacy of TA-assisted staining in the FILMP technique for managing myopic foveal retinoschisis, in comparison with ICG staining. We further demonstrated the effectiveness of TA in assisting the removal of the posterior cortical vitreous and ILM.

## Materials and methods

### Study groups

The study adhered to the Declaration of Helsinki and CONSORT guidelines and was approved by the Ethics Committee of Changsha Aier Eye Hospital. Sixty-six Chinese subjects (72 eyes) diagnosed with MF by optical coherence tomography (OCT) at Changsha Aier Eye Hospital from June 2018 to November 2020 were enrolled. The participants were divided into a TA group or an ICG group according to the staining agent used for ILM peeling during the FILMP procedure. The choice of dye (TA or ICG) was based on the operating surgeon’s established practice preference. All subjects underwent 25G PPV. Written informed consent was obtained from all participants before inclusion in the study.

#### Inclusion criteria

(i) Axial eye length ≥ 26 mm; (ii) a subjective complaint of progressive vision loss or metamorphopsia lasting more than 3 months, or demonstrated an objective decrease of more than two lines in best-corrected visual acuity (BCVA); (iii) MF diagnosed by OCT, characterized by interlayer fissures in the outer retinal layers at the macular region, accompanied by increased central retinal thickness (CRT), foveal detachment, epiretinal membrane, or lamellar hole; (iv) BCVA ≤ 0.5 (logMAR VA = log_10_1/0.5 = 0.3).

#### Exclusion criteria

(i) Severe cardiovascular or cerebrovascular diseases, liver or kidney dysfunction, or other severe systemic conditions that would make the patient intolerant to surgery; (ii) History of vitrectomy in the study eye; (iii) Presence of a full-thickness MH or retinal detachment; (iv) History of severe ocular trauma; (v) Active uveitis; (vi) Uncontrolled glaucoma; (vii) Severe leukomatous corneal opacity.

### Eye examination

All participants underwent a thorough ophthalmological evaluation before surgery, including measurement of VA/BCVA, intraocular pressure assessment, slit-lamp biomicroscopy of the anterior segment, funduscopy, OCTA (RTVue XR, Optovue), ultra-widefield fundus photography (Daytona P200T, OPTOS), and B-scan ultrasonography (AVSO, Quantel Medical). Standardized postoperative follow-up examinations were performed for all participants.

### Surgical techniques

#### FILMP with TA

**Fig. 1:**
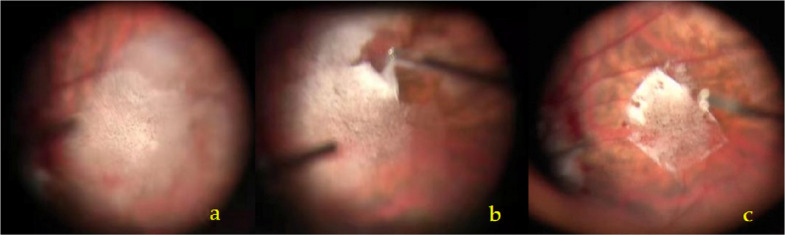
**a** The TA granules were sprinkled evenly on the retina surface of the macular area by a 27G needle with a circular *area* about 2 PD centered on the macular fovea. **b** ILM forceps were used to grasp the flap on the temporal quadrant of the TA marked area, and peel off the ILM in the form of multiple small circles. **c** One optic disc diameter of the ILM was preserved in the fovea. Excessive edge of ILM was repaired with the vitrectomy tip

Retrobulbar anesthesia was administered using a 4 mL mixture of lidocaine (5 ml: 0.1 g, Shanghai PuJing LinZhou Corp., China) and ropivacaine (10 ml: 100 mg, RenFu Pharmaceutical Co., Ltd., China) in a ratio of 5:3. A standard 3-port of 25G PPV (Constelation Vitectomy System, Alcon, USA) was performed under the Resight wide-angle viewing system (Lumera 700 + Resight 700, Zeiss, Germany). After careful removal of the central and peripheral cortical vitreous, a suspension of TA (1 ml: 40 mg, KunMing JiDa Pharmaceutical Co., Ltd., China) was injected onto the retinal surface at the macular region using a 27G needle. The TA particles were gently and evenly distributed in a concentric circle pattern approximately 2PD away from the fovea. The TA layer was maintained thin, avoiding direct coverage of the fovea to preserve visibility. Excess TA granules were aspirated with a flute needle if the deposit was too thick. The infusion valve was temporarily closed to prevent the TA from being washed away by the perfusion fluid. Following withdrawal of the surgical instruments from the sclerotomy, ILM forceps were used to grasp the ILM flap in the nasal or temporal quadrant of the TA-marked area. Because the ILM was often difficult to distinguish due to the adherence of TA granules, grasping relied heavily on surgical experience. Multiple gentle attempts at the same site were usually required. By repeatedly softly grasping and releasing at the same position, the ILM flap could be identified as it lifted slightly, guided by the TA particles. In some cases, the posterior vitreous cortex was firmly adherent to the ILM, necessitating the peeling of multiple membrane layers from the macular surface. The surgical procedure is shown in Fig. 1.

#### FILMP with ICG

Retrobulbar anesthesia and PPV surgery were performed as previously described. A 0.5% ICG solution (25 mg, YiChuang Pharmaceutical Corp., China) was injected onto the retinal surface at the macular region using a 27G needle to stain the ILM. The ICG was aspirated with a flute needle after remaining on the retina for 15 s. Once adequate staining was achieved, ILM forceps were used to grasp the ILM flap and peel a circular area approximately 1 PD away from the fovea. The peripheral retina was carefully examined for retinal breaks or lattice degeneration using scleral depression, and laser photocoagulation was applied if any abnormalities were detected. After the gas–liquid exchange, the vitreous cavity was filled with 20% C3F8, and patients were instructed to maintain a face-down position to facilitate complete absorption of the gas.

#### Define ILM and vitreous lamellae

The ILM was distinguished from the residual vitreous lamellae based on its distinct intraoperative characteristics. After TA staining, the ILM typically displayed a sharp boundary and occasional edge curling (Fig. 1c), whereas the vitreous lamellae lacked clear margins and exhibited higher viscosity. Additionally, the tactile feedback during peeling was distinct: the ILM felt more fragile and less adhesive, which facilitated its identification and separation.

#### Gas-fluid exchange

Although 20% C3F8 was used, only a two-thirds gas-fluid exchange was performed, with one-third of the intraocular fluid intentionally retained. This resulted in a final estimated C3F8 concentration of approximately 13%, which is close to the isovolumic concentration.

### Evaluation of MF recovery

During postoperative follow-up, subjects were classified into four grades based on the degree of anatomical recovery: Unattached, Improved, Near-Attached, and Well-Attached. Unattached: the neuroepithelial layers remained detached, and the CRT showed no reduction compared to preoperative measurements. Improved: remained detached, but CRT decreased compared to the preoperative level. Near-Attached: the neuroepithelial layers were mostly reattached with localized fissures, and CRT was decreased compared to preoperative values. Well-Attached: the neuroepithelial layers were completely reattached, without visible fissures, and the CRT decreased compared to the preoperative state.

### Statistical analysis

Data were analyzed using SPSS version 25.0 (SPSS Inc., Chicago, IL, USA). The Chi-square test was applied to compare categorical variables. Independent-samples t-tests and paired t-tests were used to analyse continuous variables with a normal distribution. The Mann–Whitney U test was used for non-normally distributed or ordinal (ranked) data. Data with homogeneous variance were expressed as mean ± standard deviation. Comparisons between the two independent groups were conducted using independent-samples t-tests, while paired t-tests (parametric data) were applied to compare preoperative and postoperative data within the same group. One-way ANOVA was used for comparisons among multiple subgroups. A *p*-value of ≤ 0.05 was considered statistically significant.

## Results

A total of 66 patients (72 eyes), including 19 males and 47 females, were diagnosed with MF at Changsha Aier Eye Hospital from November 2018 to June 2020. All patients underwent 25G PPV combined with FILMP. Six patients received bilateral surgery, while the remaining 60 underwent unilateral procedures. The average age was 56.16 ± 11.00 years, ranging from 30 to 73 years. The average axial length was 30.50 ± 1.96 mm, ranging from 26.19 to 34.52 mm. For statistical analysis, decimal VA was converted to logMAR VA. The mean BCVA was 1.65 ± 0.67, ranging from 0.4 to 4.0. The average CRT was 495.85 ± 206.79 μm, ranging from 126 μm to 1100 μm. The average postoperative follow-up duration was 13.89 ± 8.32 months. There was no statistically significant difference between the two groups with respect to baseline characteristics or surgical approach, as shown in Table 1.

**Table 1:** Demographics and characteristics of the patients at baseline visit

	ICG	TA	*P* Value
Number of patients *n*/(eyes)	25(28)	41(44)	
Mean age and Range (years)	58.19 ± 10.47	54.9 ± 11.24	0.23^†^
Sex (male/female)	7/18	15/26	0.56^††^
Baseline axial length (mm)	30.59 ± 2.13	30.54 ± 1.91	0.92^†^
Baseline BCVA (log MAR)	1.57 ± 0.63	1.70 ± 0.71	0.41^†^
Baseline CRT (μm)	433.70 ± 149.52	546.35 ± 231.17	0.08^†^
Posterior vitreous detachment (*n*)			
Binocular	23 (92.0%)	36 (87.80%)	
Monocular	2 (8.0%)	5 (12.20%)	0.98^††^
Posterior staphyloma (*n*)			
Binocular	22 (88.0%)	35 (85.37%)	
Monocular	3 (12.0%)	6 (14.63%)	0.64^††^
Selection of operation styles			
PPV + Gas Tamponade	3 (10.71%)	12 (27.27%)	
PPV + Gas Tamponade + Phaco + IOL Implantation	23 (82.14%)	27 (61.36%)	
PPV + Silicone Oil Tamponade	1 (3.57%)	1 (2.27%)	
PPV + Silicone Oil Tamponade + Phaco + IOL Implantation	1 (3.57%)	3 (6.82%)	
PPV + Silicone Oil Tamponade + Phaco	0 (0%)	1 (2.27%)	0.29^††^

^†^Independent samples t-test

^††^Chi-square test

A total of 61 subjects (67 eyes) completed regular follow-up, while five subjects were lost to follow-up, yielding in a follow-up loss rate of 6.94%. Three patients (3 eyes, 1 in the ICG group and 2 in the TA group) developed postoperative full-thickness MHs. The total incidence of postoperative full-thickness MHs following FILMP was 4.17%, with rates of 4.5% in the TA group and 3.6% in the ICG group. No statistically significant difference was found between the two groups (*p* = 0.082).

**Fig. 2:**
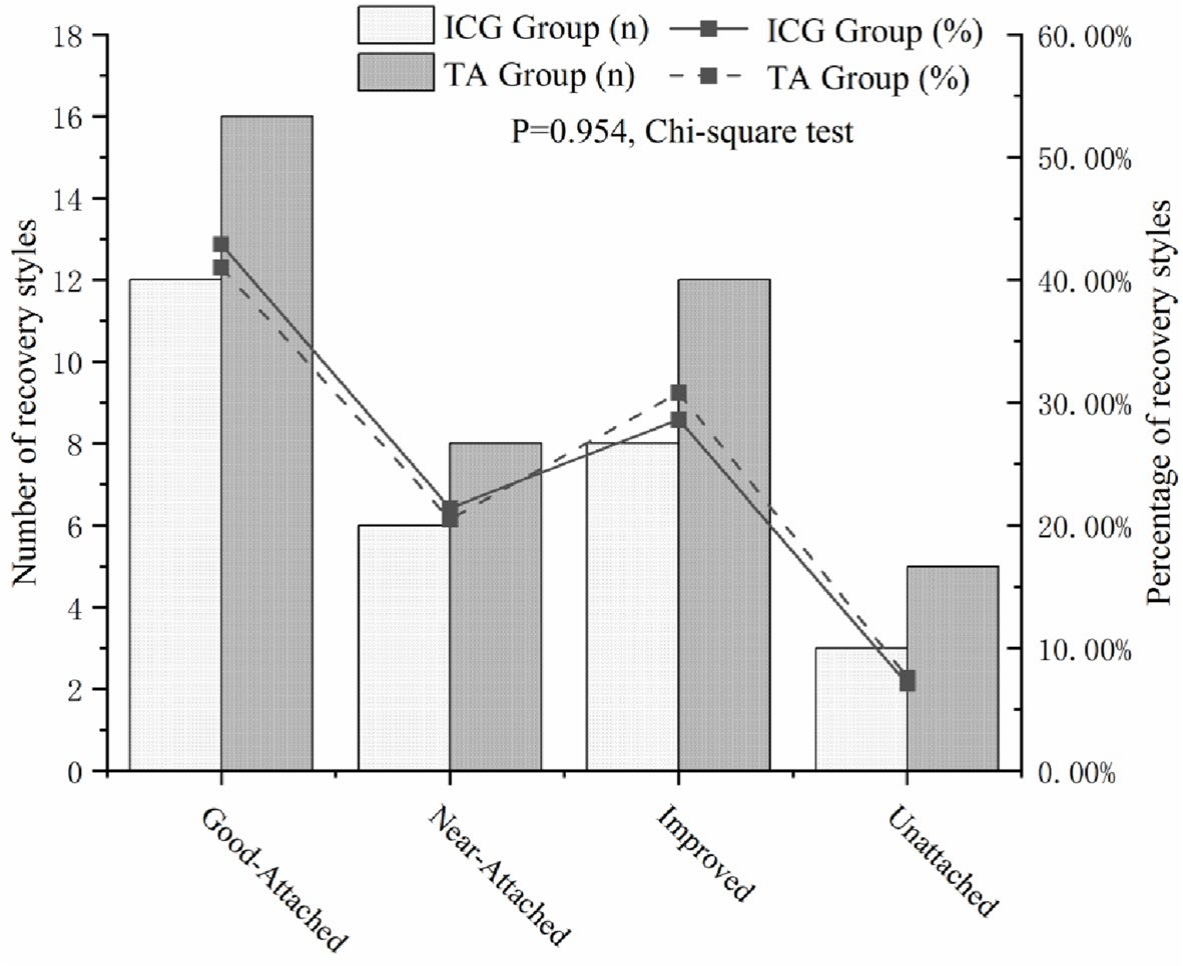
Comparison of postoperative MF recovery situation between TA and ICG group. Histograms showed the comparison of four MF recovery situations between the two groups. Line chart showed the proportions of that between the two groups. No significant difference. Comparison of postoperative MF recovery situation between TA and ICG group (*P* = 0.954)

In this study, 16 patients (18 eyes, 41.0%) in the TA group achieved well-reattachment, and 8 patients (9 eyes, 20.5%) attained near-reattachment. However, 12 patients (12 eyes, 27.3%) showed improvement, and 5 patients (5 eyes, 11.4%) remained unattached. In the ICG group, 12 patients (12 eyes, 42.9%) achieved well-reattachment, 6 patients (6 eyes, 21.4%) obtained near-attachment, 8 patients (8 eyes, 28.6%) showed improvement, and 2 patients (2 eyes, 7.1%) showed unattached. There was no significant difference in postoperative MF recovery between the TA and ICG groups (*P* = 0.954) (Fig. 2).

**Fig. 3:**
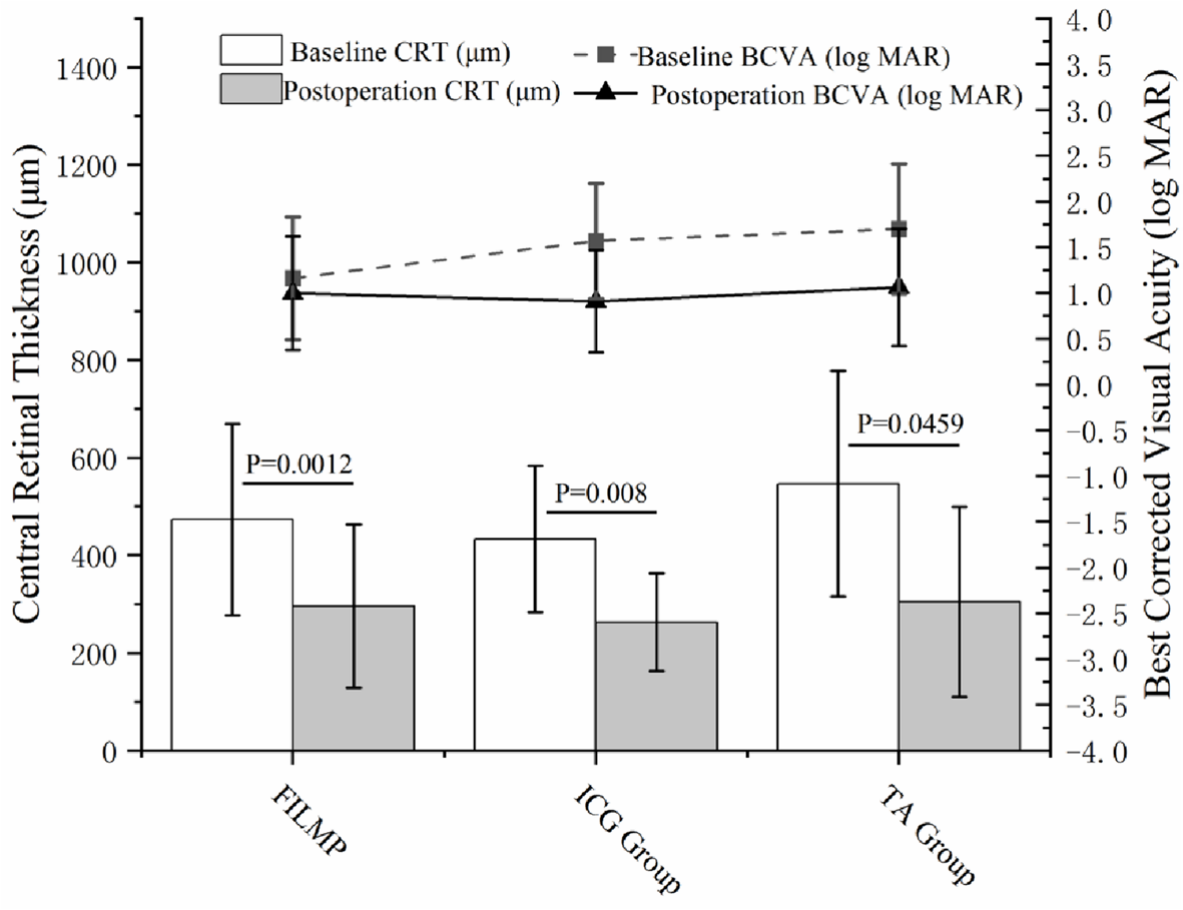
Comparison of postoperative CRT and BCVA (logMAR) between TA and ICG group. Histograms showed the comparison of four MF recovery situations between the two groups. Line chart showed the proportions of that between the two groups. No significant difference. Comparison of postoperative MF recovery situation between TA and ICG group (*P* = 0.954)

The logarithmic VA was converted to LogMAR VA for analysis. In the TA group, the mean postoperative VA was 1.06 ± 0.64, which was significantly better than the preoperative value (1.70 ± 0.71, *p* < 0.0001). In the ICG group, the mean postoperative VA was 1.57 ± 0.63, also significantly better than the preoperative value (0.91 ± 0.56, *p* < 0.0001). There was no statistically significant difference in postoperative VA between the two groups (*p* = 0.619). The difference in VA between the two groups is illustrated in the line graph (Fig. 3).

The mean postoperative CRT in the TA group was 305.15 ± 182.875 μm, significantly reduced compared to the preoperative value (526.49 ± 221.68 μm, *p* = 0.0059). In the ICG group, the mean postoperative CRT was 275.21 ± 90.57 μm, also significantly reduced compared with the preoperative value (461.89 ± 158.20 μm, *p* = 0.008). There was no significant difference in postoperative CRT between the two groups. The difference in CRT between the two groups is illustrated in the histogram (Fig. 3).

**Fig. 4:**
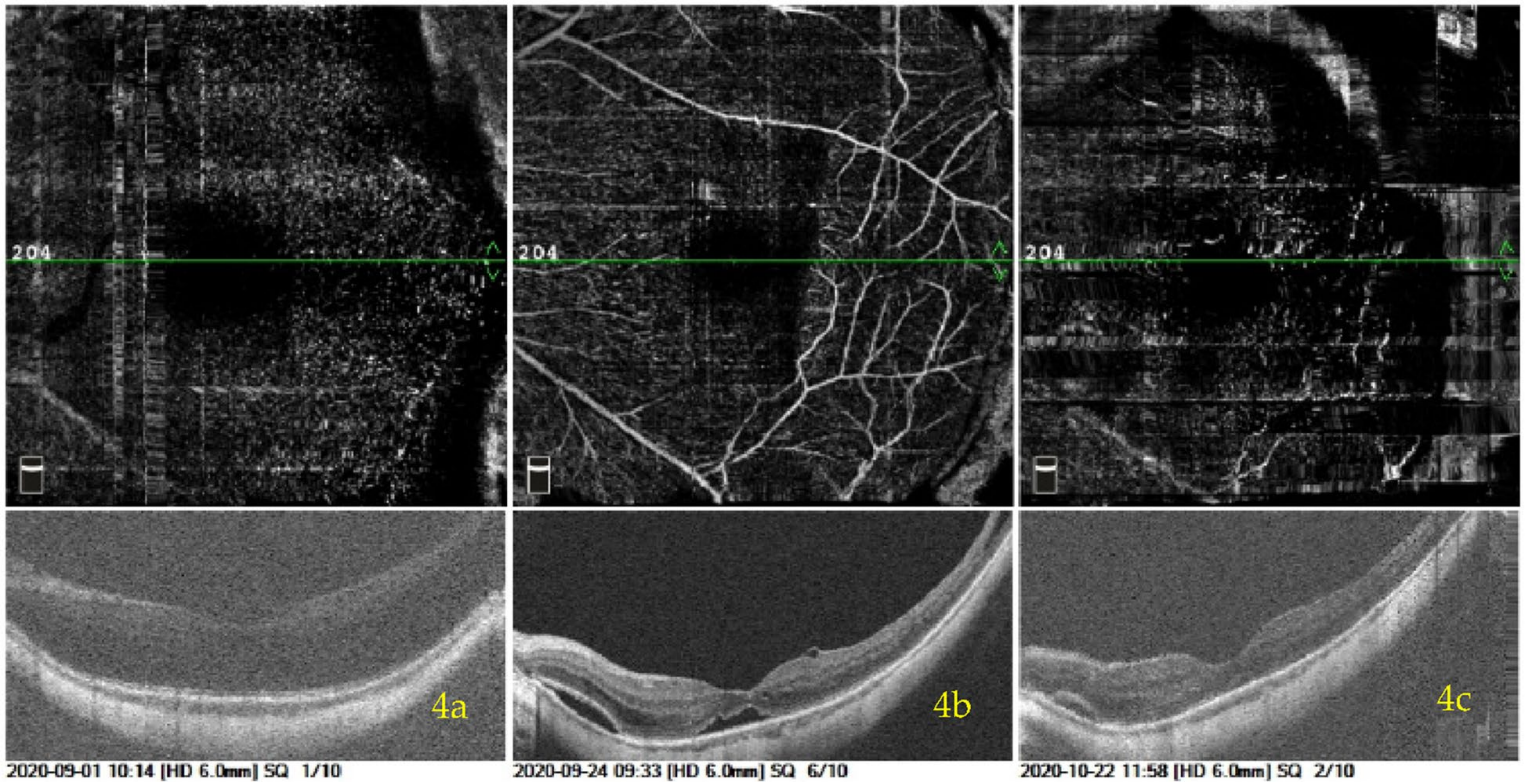
A male patient presented with decreased visual acuity in his left eye for half a year. MF with a complicated cataract and high myopia was diagnosed in his left eye by OCT. The left eye axial length was 31.48 mm. The preoperative visual acuity in the left eye was 0.05 with the best-corrected visual acuity of 0.5. The patient had normal intraocular pressure. The OCT image of preoperation is shown in (**a**). This patient underwent 25G PPV + TA-assisted FILMP with C3F8 tamponaded + Phaco + IOL implanted. The OCTA image of MF recovery saturation in 23 days after the operation was shown in (**b**). As illustrated in the OCTA image: The MF in his left eye was basically reset with two isolated small cavities found in the fovea and parafovea, respectively, and then the MF in his left eye was better reset with only one smaller cavity in parafovea in 52 days after the operation, which was shown in (**c**). The visual acuity of postoperation in his left eye was 0.8 and the CRT was 296 μm compared to 465 μm

**Fig. 5:**
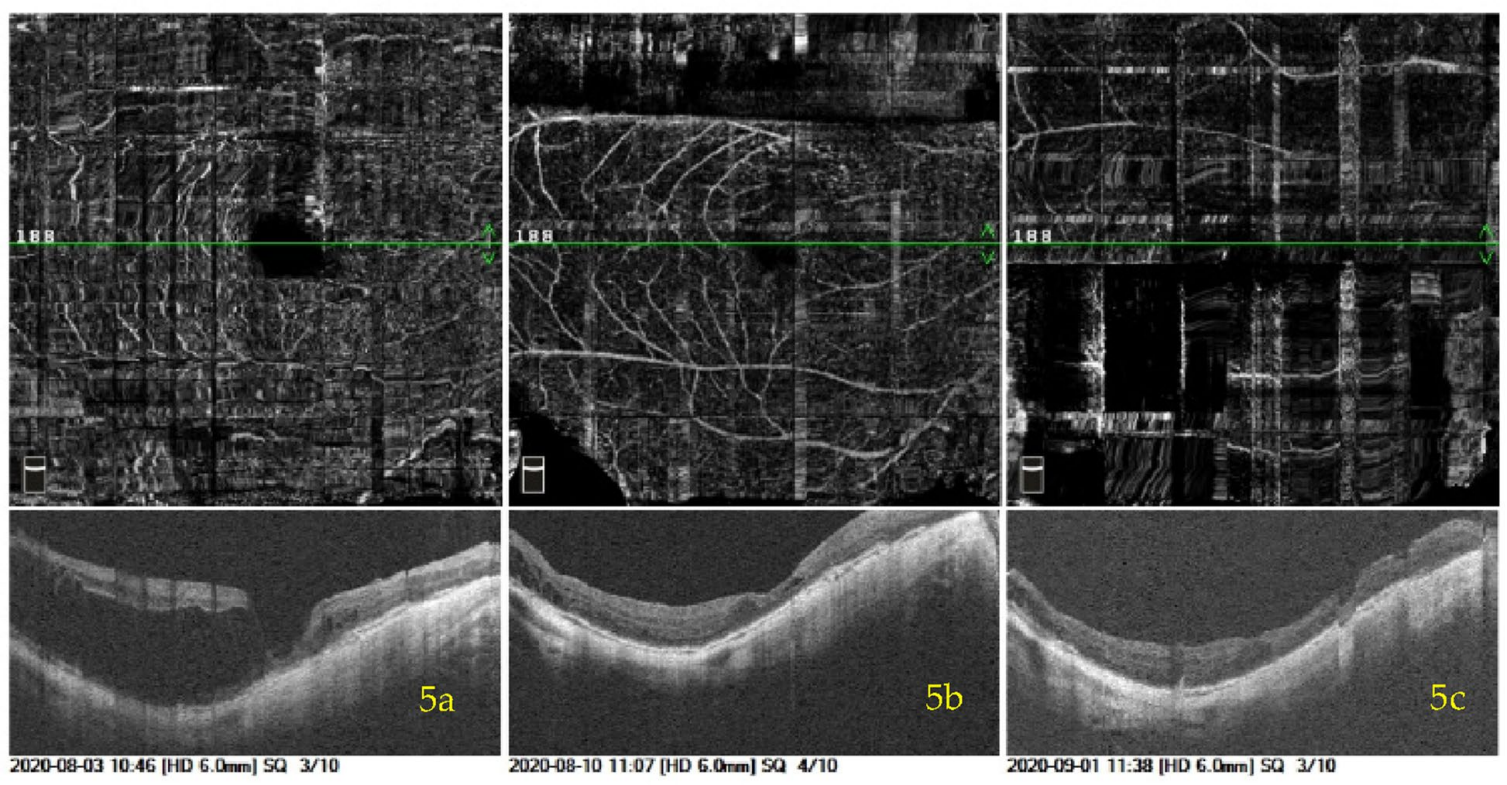
Another female patient, with reduced vision in her right eye for 3 years. She was diagnosed with MF, high myopia and complicated cataract in the right eye. The VA of preoperation in the right eye was 0.02 with best-corrected VA of 0.4. The right eye axis was 31.92 mm and the CRT was 533 μm before operation. The OCTA image of MF in her right eye before operation was shown in (**a**). She also underwent 25G PPV + TA-assisted FILMP with C3F8 tamponaded + Phaco + IOL implanted in her right eye. **b** showed the MF in her right eye was almost reattached in 6 days after surgery with a small cavity in the fovea, then the small cavity disappeared in 29 days after surgery (**c**). The CRT of postoperation was 215 μm compared to 533 μm before operation, and the VA in her right eye reached to 0.4 after operation

**Fig. 6:**
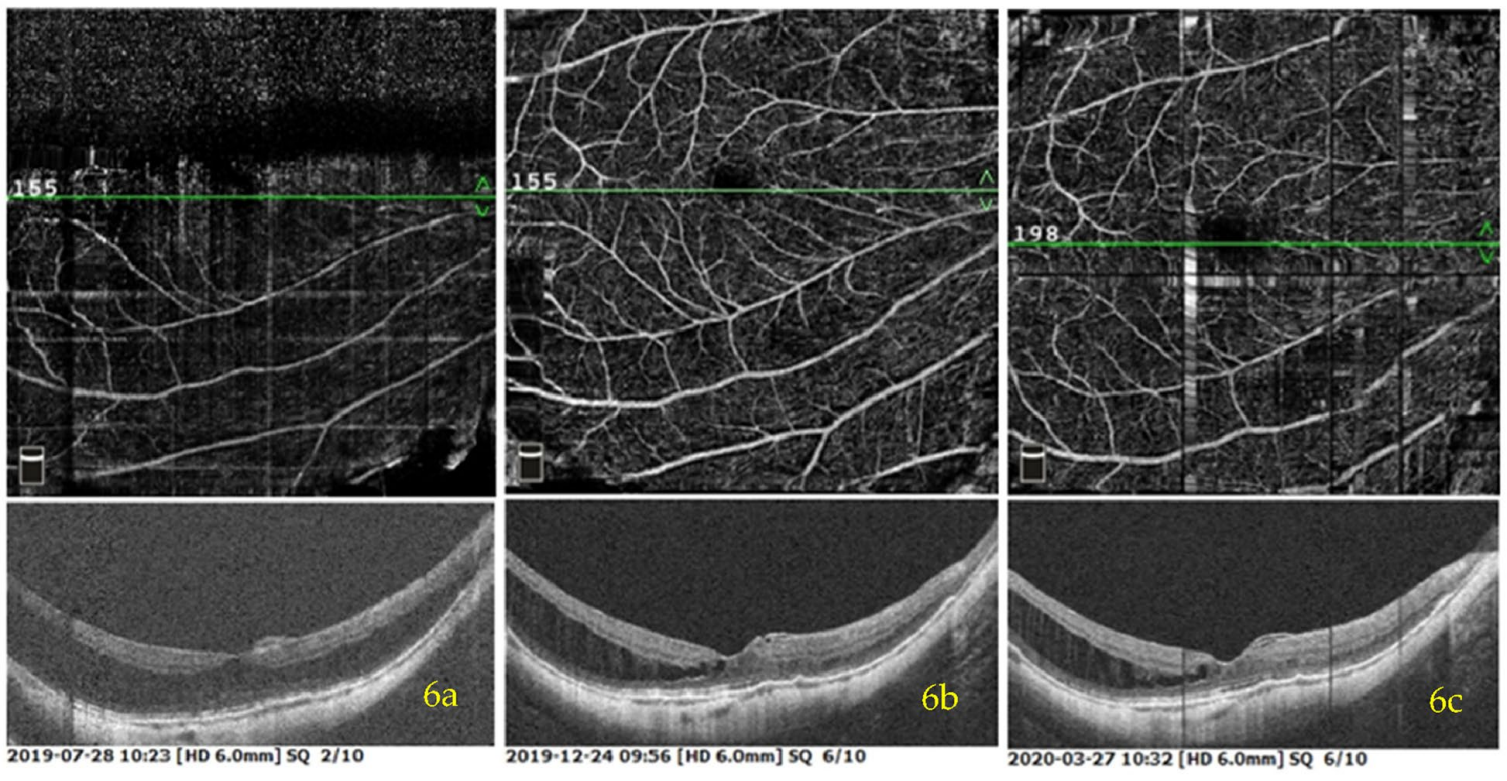
Another female patient, with reduced vision in her right eye for 40 years. She was diagnosed with MF, high myopia and cataract in the right eye. The VA of preoperation in the right eye was 0.02. The OCTA image of MF in her right eye before the operation was shown in (**a**). She also underwent 25G PPV + TA-assisted FILMP with C3F8 tamponaded + Phaco + IOL implanted in her right eye. **b** showed the MF in her right eye demonstrated partial reattachment, with one side still unadhered in 5 months after surgery, and showing no significant change in CRT in 8 months after surgery (**c**)

Three patients underwent 25G PPV + TA-assisted FILMP with C3F8 tamponaded + Phaco + IOL implantation. Details of their postoperative recovery and OCT images of MF before and after surgery are presented in Figs. 4, 5 to 6. In Fig. 4, the MF in the patient’s left eye showed notable improvement, with only a small residual schisis cavity remaining in the parafoveal region at 52 days postoperatively. This case was classified as near-attached. In Fig. 5, the patient’s right eye showed almost complete reattachment of the MF, consistent with a classification of well-attached. In Fig. 6, the MF in the patient’s right eye demonstrated partial reattachment, with persistent nonadherence on one side and no significant change in CRT in that region before and after surgery. This case was classified as unattached.

## Discussion

Globally, and particularly in China, the incidence of high myopia has been rising at an unprecedented rate each year [16]. High myopia is associated with a variety of pathological changes in the eyeball, including eye axial elongation, retinal and choroidal atrophy at the posterior pole, posterior scleral staphyloma, and MF [17]. MF is one of the most severe complications of high myopia and may progress to MH, choroidal neovascularization, or rhegmatogenous retinal detachment [18]. A regional epidemiological study conducted in China in 2012 investigated the refractive status of 5,060 university students in Shanghai and found that the prevalence of high myopia was 19.5% [19]. Evidence indicates that the incidence of MF in high myopia eyes ranges from 8% to 14.65% [2, 20]. Notably, women appear to be more susceptible to MF [21, 22]. In this study, the female-to-male ratio among patients with MF was 44:22, indicating a relatively higher proportion of female patients. The reason for this gender difference remains unclear and a possible explanation may be related to biological factors, such as endocrine differences between males and females.

A longer axial length is more likely to lead to posterior scleral staphyloma and is associated with a higher prevalence of MF. Previous studies have shown that the axial length of patients with MF ranges from 29.0 ± 0.6 mm to 30.1 ± 1.9 mm [23, 24]. In this study, the average eye axial length in subjects with MF was 30.50 ± 1.96 mm, further supporting the notion that increased axial length is an important contributing factor to the development of MF.

Posterior vitreous detachment (PVD) plays a critical role in the pathogenesis and progression of MF. In highly myopic eyes, the posterior vitreous cortex often demonstrates abnormal attachment to the retinal surface, particularly in the macular region. Incomplete or anomalous PVD may exert tractional forces on the ILM and epiretinal surface, leading to the formation of schisis cavities between retinal layers. This tangential traction is considered a key mechanical factor in the development of MF. Several studies have confirmed the association between incomplete PVD and MF. Guo observed that a high percentage of MF cases were accompanied by partial PVD and proposed that vitreomacular traction (VMT) resulting from incomplete separation of the posterior hyaloid could exacerbate retinal splitting in highly myopic eyes [25]. Similar findings were reported by Panozzo and Mercanti [26], who emphasized the role of tractional forces from a taut posterior hyaloid in the persistence or progression of myopic macular retinoschisis. In our study, a large proportion of patients presented with preoperative vitreous detachment, confirmed on OCT imaging. This suggests that persistent or incomplete vitreous detachment may contribute significantly to the pathophysiology of MF in our cohort. Importantly, even when PVD appears complete, residual vitreous cortex or lamellae may remain adherent to the ILM, especially in eyes with long axial length and posterior staphyloma, thereby continuing to exert subtle tractional stress on the retina [18].

Ikuno and colleagues reported that MF may progress from isolated schisis to foveal detachment, and eventually to lamellar and full-thickness MHs [27]. In this study, we observed cases of MF with lamellar holes preceding the development of foveal detachment. This phenomenon may be explained by tangential traction from the epimacular membrane and the ILM, which could exceed the downward force exerted by posterior scleral staphyloma. Such mechanical interactions may account for the earlier formation of lamellar holes prior to foveal detachment.

Most previous studies have reported that the incidence of postoperative full-thickness MHs following complete ILM peeling ranges from 7.9% to 28.6% [28, 29]. The FILMP technique was first proposed by Shimada [10]. In highly myopic eyes, the cystic wall of MF in the foveal region is extremely thin; thus, complete removal of the ILM over the fovea may lead to cyst rupture and subsequent MH formation. To prevent this complication, Shimada et al. introduced a modified ILM peeling technique that preserves the ILM over the fovea, thereby minimizing cyst wall damage and reducing the risk of postoperative MHs. Subsequent studies have confirmed the effectiveness of FILMP. Shirak et al. [29] reported 26 cases of high myopic-associated MF treated with FILMP, with no postoperative MHs observed. Elsewhere, Tian et al. [30] reported 18 cases of high myopia-associated MF treated with FILMP, among which one patient developed a postoperative full-thickness MH. In our study, three subjects developed full-thickness MHs after FILMP, resulting in an incidence rate of 4.17%. All three cases were complicated by lamellar MHs prior to surgery. Although retinal reattachment was achieved postoperatively, the lamellar holes progressed to full-thickness MHs during follow-up, which may be attributed to severe posterior scleral staphyloma. Although the retina was reattached over the staphyloma, the retinal tissue may have been insufficient in length to close the MH, leading to its persistence. Therefore, in cases of high myopic-associated MF complicated by severe posterior scleral staphyloma, vitrectomy combined with ILM peeling alone may be insufficient for anatomical repair. Additional procedures, such as posterior scleral reinforcement, may be required to address the underlying scleral ectasia [31].

In this study, although the mean postoperative BCVA improved compared with preoperative values in patients with MF, some patients, particularly those without significant cataract, did not experience noticeable visual improvement after surgery. This suggests that ILM peeling may not always lead to functional gains in certain MF cases, possibly due to pre-existing atrophy of the choroid or retinal neuroepithelial layers, even if anatomical restoration of the macula is achieved [32].

All MF subjects in this study underwent FILMP using 25G PPV. Because 25G intraocular surgical instruments are shorter than 23G instruments, a vertical puncture incision was made at the 10 o’clock position, after which the cannula was removed. This allowed forceps and vitrectomy instruments to directly enter the eye through the incision without the cannula. This approach not only reduces the possibility of the TA particles covering the macular area being washed away by the perfusion fluid through the incision, but also eliminates the limitation imposed by the shorter length of the 25G instrument. Consequently, patients with an eye axis length of 30 mm or more can still safely undergo 25G minimally invasive vitrectomy.

Controversy remains regarding the potential retinal toxicity of intraocular ICG. However, due to its strong affinity for the ILM, many surgeons continue to use ICG. Evidence indicates that the use of ICG or other dyes for ILM staining during vitrectomy may be toxic to the retina [33, 34]. In contrast, TA is considered safe and non-toxic for intraocular use and has been widely applied in vitrectomy procedures [35]. Moreover, ICG is often ineffective in some cases of MF with tightly adhered posterior vitreous cortex, in which the ILM cannot be adequately stained. In such cases, TA granules could adhere to and delineate the posterior vitreous cortex in the macular area of high myopia eyes [36], thereby facilitating its removal. Subsequently, TA can be reapplied to stain the ILM for peeling, without concerns about potential toxicity from repeated application.

Although BBG is widely recognized as a safer alternative to ICG for ILM staining, it was not employed in this study. The primary reason is that BBG exclusively stains the ILM and provides limited visualization of the posterior vitreous cortex or epiretinal membrane, which are often firmly attached in highly myopic eyes with foveoschisis. By contrast, TA enhances visualization of both the vitreous cortex and ILM, allowing surgeons to identify and peel multiple tractional layers more effectively. Therefore, TA may serve as a more versatile and safer agent for macular surgery in such complex cases. Further comparative studies of BBG and TA are warranted to explore their respective roles and limitations.

However, TA-assisted FILMP requires considerable surgical expertise and is associated with a distinct learning curve. Additionally, because TA particles may obscure the entire macular area, the underlying retinal tissue becomes invisible during the procedure. Therefore, the surgeon must precisely localize the fovea preoperatively to avoid accidental damage. Previous study [37] have reported that dilution of TA can improve the visibility of the ILM by allowing a thinner and more uniform particle layer on the retinal surface. This technique facilitates clearer identification of the ILM margins and minimizes the risk of inadvertent foveal injury. Especially in highly myopic eyes with fragile foveal structures, the use of diluted TA may help reduce the likelihood of iatrogenic trauma during membrane peeling.

## Limitations

This study has several limitations. The sample size was relatively small and the follow-up duration was limited, which may affect the generalizability of the findings. In addition, intraoperative details such as the number of attempts required to grasp the ILM and the frequency of ILM shredding were not systematically documented, limiting the evaluation of surgical fluency. Moreover, subtle postoperative changes, including disorganization of the retinal inner layers (DRIL), were not specifically assessed in our follow-up protocol. Future prospective studies with larger cohorts, longer follow-up, and more detailed intraoperative and postoperative assessments are needed to further clarify the relationship between surgical technique, retinal microstructural changes, and long-term visual outcomes.

## Conclusion

This study shows that TA can effectively label the vitreous cortex, epiretinal membrane, and ILM. In cases of MF, TA is safe, harmless, non-toxic, convenient to use, and it can avoid potential retinal toxicity associated with repeated use of ICG staining. In this study, the TA-assisted FILMP surgery achieved comparable outcomes to ICG-assisted FILMP in terms of postoperative CRT, BCVA, anatomical recovery of MF, and the incidence of postoperative full-thickness MHs. These findings support the notion that TA may serve as an effective alternative to traditional dye staining in macular surgery. Further large-scale, prospective studies and randomized controlled trials are warranted to clarify its superiority.

## Data Availability

The data used or analyzed during this study are available from the corresponding author on reasonable request.

## Abbreviations

MF: Myopic foveoschisis
ERM: Epiretinal membrane
ILM: Internal limiting membrane
MH: Macular hole
PPV: Pars plana vitrectomy
FILMP: Fovea-sparing ILM peeling
ICG: Indocyanine green
RPE: Retinal pigment epithelium
TA: Triamcinolone acetonide
BBG: Brilliant blue G
OCT: Optical coherence tomography
BCVA: Best-corrected visual acuity
CRT: Central retinal thickness
IOL: Intraocular lens
PVD: Posterior vitreous detachment
VMT: Vitreomacular traction
DRIL: Disorganization of the retinal inner layers
